# Medical students as peer tutors: a systematic review

**DOI:** 10.1186/1472-6920-14-115

**Published:** 2014-06-09

**Authors:** Annette Burgess, Deborah McGregor, Craig Mellis

**Affiliations:** 1 Sydney Medical School - Central, The University of Sydney, Building 63, level 4, Royal Prince Alfred Hospital, Missenden Road, Camperdown, New South Wales 2050, Australia

## Abstract

**Background:**

While Peer Assisted Learning (PAL) has long occurred informally in medical education, in the past ten years, there has been increasing international interest in formally organised PAL, with many benefits for both the students and institutions. We conducted a systematic review of the literature to establish why and how PAL has been implemented, focussing on the recruitment and training process for peer tutors, the benefits for peer tutors, and the competency of peer tutors.

**Method:**

A literature search was conducted in three electronic databases. Selection of titles and abstracts were made based on pre-determined eligibility criteria. We utilized the ‘AMEE Peer assisted learning: a planning and implementation framework: AMEE Guide no. 30’ to assist us in establishing the review aims in a systematic review of the literature between 2002 and 2012. Six key questions were developed and used in our analysis of particular aspects of PAL programs within medical degree programs.

**Results:**

We found nineteen articles that satisfied our inclusion criteria. The PAL activities fell into three broad categories of teacher training, peer teaching and peer assessment. Variability was found in the reporting of tutor recruitment and training processes, tutor outcomes, and tutor competencies.

**Conclusion:**

Results from this review suggest that there are many perceived learning benefits for student tutors. However, there were mixed results regarding the accuracy of peer assessment and feedback, and no substantial evidence to conclude that participation as a peer tutor improves one’s own examination performance. Further research into PAL in medicine is required if we are to better understand the relative impact and benefits for student tutors.

## Background

There has been much written about the use of Peer Assisted Leaning (PAL) and the associated cognitive, pedagogical, attitudinal, social and economic benefits associated with utilising peer tutors [1-3]. While PAL has long occurred informally in medical education, in the past ten years, there has been increasing international interest in formally organised PAL, as reflected in the growing body of published literature [4,5].

PAL is of interest because of the well documented benefits at several levels. For the institution, PAL can alleviate faculty teaching burden [6] offering a potential resource saving measure, to accommodate the increasing number of medical students undertaking early clinical activities, in what was previously considered pre-clinical years [7,6]. PAL can assist the institution to meet external expectations for medical graduates to achieve competency and experience in both teaching and assessment, and may help to instil a life-long culture of teaching [7]. PAL can also address specific gaps within the curriculum [8], providing additional student support in preparation for assessments [9].

For the peer tutor, there remains some uncertainty as to whether participation in PAL actually improves examination performance [10]. However it has been asserted that PAL offers a valuable method of enriching students’ learning experience [11]. There are many documented benefits to having medical students learn how to teach and assess; and in being provided with opportunities to practice these skills [12]. As medical graduates, they are expected to be skilled in life long learning, an attribute that PAL activities can help students develop through gaining competence in reflecting and expanding on their own knowledge [13]. As medical practitioners and educators, they will also be expected to supervise, teach, facilitate, assess and provide feedback to colleagues, and contribute to the teaching of future generations of medical students. Evidence suggests that participation in PAL is an effective and efficient way to introduce and foster these core professional skills that may not be included in formal medical or health care professional curricula [4-6,14]. PAL is said to provide leadership, coaching, learning skills training, enhance confidence and intrinsic motivation, and may also promote an interest in academic careers [6].

While descriptions of PAL in the literature are abundant, there remains limited literature about formal attempts to facilitate the development of medical students’ teaching skills [14]. There are many considerations when establishing and reporting on a peer teaching program, particularly those relating to the tutor [5]. It is evident that there is little consensus on the optimal recruitment process, training needs, and the teaching competencies expected of medical student tutors [7]. In view of this uncertainty, we have undertaken this systematic literature review, and have concentrated on the outcomes for the peer tutors, and the forementioned areas that may affect such outcomes.

### Review objectives

We utilized the ‘AMEE Peer assisted learning: a planning and implementation framework: AMEE Guide no. 30’ to assist us in establishing the review aims in a systematic review of the literature between 2002 and 2012 [5].

This review aims to answer the following questions:

1. Why is PAL being implemented by medical schools?

2. What PAL activities are peer tutors involved in?

3. What is the recruitment process for peer tutors?

4. What is the training process for peer tutors?

5. What are the effects of PAL participation on the attitude, knowledge and learning outcomes of peer tutor participants?

6. How has the competency of peer tutors been determined?

## Methods

### Definitions

Peer-assisted learning (PAL) has been defined as “People from similar social groupings who are not professional teachers helping each other to learn and learning themselves by teaching” [1]. Although broader, more inclusive definitions exist [5,15,16], for the purpose of this literature review, we have chosen to focus on the “teaching” aspect.

Medical students are defined as those enrolled in tertiary programs who will qualify as medical practitioners. Tutors are the student teachers or assessors. Tutees are the students being taught or assessed by their peers.

The following databases were searched: Medline: via Pubmed, Web of Knowledge and ERIC databases. The search strategy comprised combinations of the search terms “medical education”, “medical education, undergraduate”, “medical education”, “peer-assisted learning”, “peer-teach*”, “peer-tutor”, “peer-assessment” and “peer-evaluation”. The search was limited to original articles published in the past decade (2002–2012).

### Inclusion criteria

Included were articles reporting on undergraduate or graduate entry medical education (ie, ‘medical students’, as defined above). Articles were only included where formal peer teaching and/or assessment of clinical skills and procedural skills programs were reported. Articles documenting programs where students receive instruction how to teach and/or assess were also included. Only articles written in English were included.

### Exclusion criteria

The following results were excluded in this review:

•Peer evaluation where students observed professionalism.

•Peer evaluation completed at the end of a course.

•Peer assessment performed by peers following a student presentation made as part of a course.

•Programs involving medical graduates, for example, residents acting as the peer-tutor to training medical students.

•Studies involving nursing and the other health sciences.

•Studies where the same PAL activity had been reported previously.

**Figure 1:**
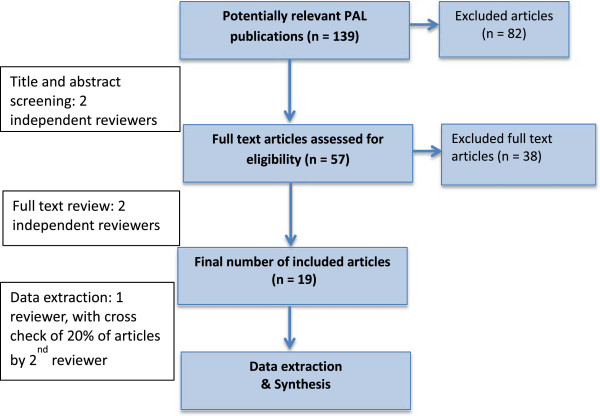
Flowchart of article search process in the systematic review of the literature on the implementation of peer assisted learning in medical schools published 2002 - 2012.

Our literature search yielded 139 potential publications on PAL in undergraduate medical program (see Figure 1 for our complete search and study selection strategy). Following an initial review for relevancy by AB and DM and removal of duplicate results we had a total of 57 citations. These papers were then independently appraised for relevance by two authors (AB and DM). This left 19 papers deemed relevant to our systematic review, and each complete manuscript was then analysed by considering the six previously mentioned questions [5].

## Results

This systematic review includes a total of 19 papers. Additional file 1: Table S1 describes the detail of the 19 papers, in terms of PAL implementation background; the seniority of PAL participants; recruitment processes; training procedures; and tutor outcomes.

Eighteen of the studies were scattered across eight different countries (one study did not identify the institution or country), with Germany representing more than a quarter (5/19) of the studies, at five different universities. Australia was the next most commonly represented country, with four studies at three different universities. Other countries represented included England with three studies at three different universities; Scotland, with two studies at the same university, and one study each in Ireland, Canada, USA, and Malaysia.

### Why is PAL being implemented by medical schools?

Several papers reported practical reasons and staff resource issues being linked to their decision to explore PAL [4,17-24]. Three of these papers, as well as several others, identified the need to develop medical students’ skills in teaching and assessment as a consideration in their implementation of PAL [4,18,23,25-29].

### What PAL activities are peer tutors involved in?

The activities reported fell into three broad categories: Teacher training programs; Peer assessment; and Peer teaching. However, the degree and level of training provided within the peer assessment and peer teaching programs varied greatly.

#### Teacher training

Two articles [25,29] reported on a standalone teacher training programs for medical students.

#### Peer teaching

The majority of PAL articles found (12/19) reported on Peer Teaching, with a vast amount of variation in teacher training being provided within, or as an extension of the implemented program [4,17,19-24,26,28,30,31].

#### Peer assessment

Five articles [18,27,32-34] reported on Peer Assessment, again with varying degrees of teacher training. Two of these articles reported on peer feedback alone [33,34] and although these are categorized as peer assessment, students were not assessing their peers, but providing feedback only to their peers during assessment activities.

#### PAL subject and discipline focus

All but one of the peer assessment and feedback activities were within OSCEs, covering topics including history taking and communication skills; physical examination skills; and procedural skills. There was one peer assessment article where the skill being assessed was Basic Life Support [27]. Peer Teaching activities included history taking and communication skills; physical examinations skills, including Rheumatology Gait Arms Legs Spine (GALS), neurological examination and lumber puncture, musculoskeletal ultrasound interpretation, ECG interpretation, episiotomy; and procedural skills in internal medicine basic skills.

#### PAL participants

The majority (12/19) of the studies involved “near peer” teaching, with the more senior students responsible for teaching or assessing the junior students; five of the studies involved direct peer to peer (in the same year); and for the two teacher training programs this was irrelevant, although both were only offered to senior students.

### What is the recruitment process for peer tutors?

Although the tutor recruitment process was not always clearly reported, for the majority (at least 11/19) of PAL programs, tutor participation was voluntary, as an additional activity, open to all students within a particular cohort. Only one program was embedded in the core curriculum requiring compulsory participation for all student tutors [33], where students were required to give direct peer to peer feedback during an OSCE.

In one project [24], where tutor participation was voluntary, there was an additional interview selection process where tutor experience, leadership qualities, accomplishments during clerkships and the level of motivation served as criteria for selection. In the study by Glynn et al., [17], recruitment was via a voluntary process, though students needed to have successfully completed particular clinical placements and academic assessments. Reported motivation for volunteering as tutors include both extrinsic and intrinsic rewards. Both Weyrich et al. [24] and Nikendei et al. [31] reported that peer tutors received financial compensation for participation. Reported intrinsic rewards include an enjoyable experience of helping others, gaining new insights and understanding of assessment processes; a greater understanding of the topic; and developing skills in teaching and assessment.

### What is the training process for peer tutors?

Peer teaching and facilitation training was reported as a standalone program [25,29], or as a tutor preparation component of a peer teaching or peer assessment program. However, in two reports, no preparatory tutor training was provided [17,32]. Training and preparation for tutors varied in terms of content and duration. Each was specific to facilitation/teacher skills training; or content specific knowledge and skills; or a combination of both. The majority of papers described tutor training that included facilitator skills training as well as content specific training [4,18-22,26,28,30]. The practical training being provided was appropriate to the role the tutor would be performing; the qualities expected of a teacher; how to give feedback; confidentiality; marking criteria; administrative process; specific subject knowledge and skills required; and discussion around what topics may arise during the PAL session. Three papers reported only on facilitation skills training without content specific training, before having students teach peers in clinical and procedural skills [23,24,34]. Teacher training programs focussed mostly on the basic principles of teaching, including theory and practical training [25,29].

The expected time commitment for tutor training varied greatly, with as much as 18 hrs across multiple (6) sessions [25], and 10 hrs across 5 workshops [29]. Silbert and Lake [23] reported that senior student tutors were required to attend a modified Teaching on the Run course, encompassing two 3 hour interactive workshops, before taking part in teaching junior students examination techniques. With an emphasis on the clinical setting, the modules for these three programs were similar, focusing on communication, skills teaching, assessment and providing of feedback. Weyrich et al. [24], also reported on a similar time commitment for training, with students being required to attend two 3 hour consultant led training sessions, although it appears that this training had more clinical skills and content knowledge focus, with the training being carried out in the skills lab and technical skills being demonstrated by the consultant and repeatedly practiced by the tutors [24].

Interestingly, one article reported peer assessors being sent to a formal external instructor course incorporating teacher training and skills training [27]. Some studies, however, reported minimal tutor training prior to the actual PAL activity. For example, only one hour of training was delivered for tutors in preparation for peer assessment in an OSCE session detailing the OSCE question, marking criteria, examination and feedback techniques [25]. One paper reported that the only “training” provided was through observation of a faculty member providing feedback to a student, with the peer tutor needing to then provide additional feedback to the same student [33]. Some training involved aspects of self-directed learning, for example completing a literature review; or practicing skills with each other [22,26,30]. In a few cases, additional support material, such as tutor manuals were provided [4,31]. Others described processes for ongoing content and educational support [20].

### What are the effects of PAL participation on the attitude, knowledge and learning outcomes of peer tutor participants?

The student tutor self-perceived benefits fell into two broad categories:

•Development of professional attributes.

•Development in the understanding of knowledge content.

#### Development of professional attributes

Many of the studies found student tutors considered the activity useful for their future careers in developing professional attributes. These included an increased understanding and awareness of facilitation, teaching, assessment and feedback techniques [4,18,23,25,30,31]; development of leadership qualities [24]; ability to admit uncertainty [20]; development of confidence; [20,24] fostering a willingness to contribute to the education of others [18,24]; and autonomy in learning [18]. However, some peer tutors felt awkward in providing feedback [33].

In the two teacher training programs, the program was highly valued by students, although Merglen [29] did not report on any specific areas of perceived or measurable benefits to student participants. Burgess et al. [25] expanded on the students’ perceived benefits to include an increased appreciation of educational theory and practice, increased perceived ability to plan learning activities; increased perceived ability to provide effective feedback to peers; and feeling valued by senior academic staff who ran the course.

#### Development in the understanding of knowledge content

Several papers identified perceived opportunity by peer tutors for revision of knowledge; opportunity to reflect on their own knowledge gaps and a deeper level of understanding of content [18,20,23]. In teaching others physical examination skills, peer tutors reported increased confidence in examination skills themselves [30]. Students also found it educationally useful to formulate and deliver feedback to peers [33]. Two papers identified the relaxed environment for the tutors as being conducive to learning [20,24]. Some studies did not consider the perceived, subjective benefits to the peer tutors [19,21,27], and one study reported only the “enjoyment” of student tutors [26].

#### Objectively measured benefits for tutors

Knowledge acquisition by student tutors was measured in two studies using student tutors’ examination performance, which produced conflicting results. Knobe et al. [21], reported that student tutors, teaching shoulder ultrasound interpretation, to students within the same year achieved significantly higher results over all in their MCQ and OSCE examinations [21]. However, Nestel and Kidd [4], reported no benefit to student tutor knowledge acquisition as determined by a communications skills examination performance when results of tutors were compared to non-tutors.

### How has the competency of peer tutors been determined?

#### Marking ability of student tutors

Only one article (Bucknall et al., [27]) reported testing teacher competencies of the peer tutors prior to participation in PAL activities, and interestingly, this is the only article reporting accuracy in student marking [27]. Reiter et al. [32] reported that where students were examined by tutors in an OSCE, student examiners awarded significantly higher marks than the faculty examiners. Similarly, Burgess et al. [25] found that peer assessors could not competently determine a global mark in OSCE practice examinations. Bucknall et al. [27], however, found a good level of agreement in marking between student and faculty examiners in a basic life support end-of-course test, with student examiners being more cautious than faculty to award a pass.

#### Quality of feedback

Quality of the feedback as assessed by the tutees was considered in one paper [32], and it was considered by students to be superior to faculty feedback. The PAL activity implemented by Brazeau [33] required students to give feedback to peers and was regarded as educationally useful by both faculty and students.

## Discussion

### Rationale by medical schools for the implementation of PAL activities

In isolation, it is ethically difficult to defend offering PAL activities as a result of staff resource issues without offering demonstrated advantages or at least no disadvantages to students. All studies sought to show that student learners were not disadvantaged by the new PAL initiatives. PAL may be seen by the institution as a means to provide additional student support in preparation for high stakes summative assessments [18].

It is unsurprising that many papers identified the development of students’ teaching and assessment skills as a reason for implementing PAL activities. These are professional attributes required in future employment that are increasingly recognised by universities and medical councils as professional attributes formally listed as graduate competencies by universities and medical councils [7].

### The format of PAL activities involving peer tutors

The most popular form of PAL appears to be peer teaching activities, rather than peer assessment activities. The assessment activities were generally in the form of formative OSCEs, which may be attributed to the extensive resources required to run an OSCE, and the resource savings that peer facilitation offers. It is interesting that the majority of papers reported “near” peer teaching, with the senior students teaching, or assessing, their junior colleagues.

### Recruitment of tutors

Voluntary student tutor recruitment was the most common form of recruitment. There appears to be some debate around how recruitment of peer tutors should take place. Ten Cate and Durning [6] suggest that PAL participation should be “part of the regular mandatory programme” in order to increase efficiency. However, Wadoodi & Crosby [35] suggest that participation as a peer tutor should be voluntary, and should not exclude potential volunteers on the basis of academic performance. This is because the aim is not to “use” students as a resource saving, but to implement a mutually beneficial educational activity for both tutees and tutors. It can also be argued that students of greater academic standing may not necessarily be in a better position to identify and assist tutees with difficulties in understanding, as skills in facilitation are important and not determined by academic ability [35]. Further, there is evidence to suggest that the experience of participating as a peer tutor is more likely to benefit poor performing students and therefore they should not be excluded [36].

Two of the studies offered monetary payment to students. Ross & Cameron [5] suggest that where participation is voluntary, the intrinsic and extrinsic rewards may have considerable bearing on recruitment and retention of PAL tutors, but suggest that the intrinsic rewards negate the use of any extrinsic rewards.

### Teaching and facilitation training

Although no training was offered in one of the OSCE peer assessment activities, the authors suggest that tutors may have received better assessments if they had formal training [32]. Typically a teaching role entails teaching new content, helping tutees learning knowledge or new skills; or providing assessment with feedback. PAL tutors are typically not trained teachers or experts in the topic being instructed. Generally, they have less expansive knowledge of the subject matter, and less developed teaching skills than expert tutors [15]. Therefore, it is asserted that tutor training should be aimed at developing both teaching skills, as well as content specific knowledge [35]. Ross & Cameron [5] suggest that the amount of training should be dependant upon the requirements of the tutor activity, and suggest a needs analysis in order to develop a specific training package. The latter may include pre-reading, formal training, and assessment of competence of content knowledge, and/or teaching ability [5,37].

While wider educational literature suggests that tutor training may enhance the outcomes for tutees [1,38,39], there is insufficient evidence reported within medical education literature to confirm this [5].

### Student outcomes

#### The student tutor self-perceived benefits

Tutor self-perceived benefits included skills in professionalism and development in the understanding of knowledge. The findings here are supported by wider PAL literature where it is commonly reported that it is the student tutors who gain most from PAL interventions [1,40]. Evidence suggests that preparing to teach, teaching, assessing, providing feedback to peers and reflecting offer both cognitive and non-cognitive benefits to tutors [41]. By participating in PAL, tutors are driven to engage, analyse and verbalise what they know, and to realise and address their own knowledge gaps [1,38]. A highly effective enquiry based learning experience for students is provided through peer teaching, where the act of teaching another student involves using knowledge and skills to engage with another student, promoting a deeper engagement with learning [42]. Although some studies reported student concerns about assessing their peers and providing feedback, it provides and important education tool in developing these professional competences [43].

#### Objectively measured benefits for tutors

We identified one study [21], reporting objectively measured improvement in knowledge acquisition due to peer tutoring, and one study that was not able to identify any increase in knowledge acquisition to peer tutors. Indeed, currently there is insufficient evidence in the PAL literature to determine whether or not participation as a tutor in peer assessment does actually improve student performance [10].

### Measurement of student tutor competencies

#### Marking ability of student tutors

Two studies reported peer assessors as being more lenient markers than academic assessors [25,32], while one study found academic assessors to be more lenient markers that peers [27]. Although it can provide a valuable method of enriching students’ learning experience, there are mixed reports regarding the accuracy of peer assessment [11]. The process can lack objectivity and is subject to bias [44,45].

#### Quality of feedback

Although the quality of the feedback as assessed by the tutees was superior to faculty feedback in one paper [32], there was no objective measure to determine the accuracy of this feedback. Similarly, the study by Brazeau et al. [33] included no objective measurement regarding the accuracy of peer feedback.

### Limitations

It is possible our search strategy may have missed some published papers. We felt that since the popularity of PAL activities within medical education has only occurred over recent years, that the past 10 years of data would capture most of the relevant published data. We also confined our search to articles written in English. However, it is unlikely that we have missed a substantial number of such publications. Although articles from 2013 onwards were not included in the review, a recent search of the period 2013–2014 has found four relevant papers, with similar outcomes to those reported in our paper.

## Conclusion

The rise in international interest in PAL appears to be a consequence of the global increase in medical student intake, limited teaching resources, and an emergent reference to teaching and assessment capabilities as graduate competencies. The mixed results regarding accuracy of peer assessment and feedback warrants further research and investigation using objective measures. Although results from this review suggest that there are many perceived learning benefits for student tutors participating in PAL activities, no substantial evidence was found to conclude that participation as a peer tutor improves one’s own examination performance. It also appears that there is variation in recruitment processes, and duration and content of tutor training, with little evidence of related effects on student tutor outcomes, warranting further investigation.

## Competing interests

The authors declare that they have no competing interests.

## Authors’ contributions

AB: literature review concept design, analysis and interpretation of data, drafting of manuscript. DM: analysis and interpretation of data, drafting of manuscript. CM: analysis and interpretation of data, drafting of manuscript. All authors read and approved the final manuscript.

## Pre-publication history

The pre-publication history for this paper can be accessed here:

http://www.biomedcentral.com/1472-6920/14/115/prepub

## Supplementary Material

Additional file 1: Table S1 Context, tutor recruitment, tutor training, and tutor outcomes within PAL implementation. Click here for file
