# Supplementary material for: Medical students as peer tutors: a systematic review
Source: BMC Med Educ. 2014 Jun 9;14:115. doi: 10.1186/1472-6920-14-115 (PMC4237985; doi:10.1186/1472-6920-14-115)
Supplement: Additional file 1: Table S1 — Context, tutor recruitment, tutor training, and tutor outcomes within PAL implementation. [file 1472-6920-14-115-S1.docx]

**Table 1. Context, tutor recruitment, tutor training, and tutor outcomes within PAL implementation**

| Author | **Cat** | **The PAL project** | **PAL Tutors** | **PAL tutees** | **PAL Tutor training** | **Plan of activities during the PAL interaction** | **Benefits for tutors/assessors**  **& outcomes.** |
| --- | --- | --- | --- | --- | --- | --- | --- |
| Reiter et al, 2004 | **PA** | McMaster University, Canada.  MD clinical clerks as assessors of junior students in an OSCE (for two stations). | Six final year medical students as examiners.  Voluntary participation.  Final year students invited to be peer examiners by email. The first six to respond chosen. | 128 first year students in the OSCE. | No dedicated training described.  Future examiner training recommended. | At two of ten test stations, one clinical clerk and one faculty member or resident evaluated each examinee. | Student examiners assigned significantly higher marks than the faculty examiners.  The quality of feedback from peer examiners was rated the highest by the student examinees.  Having students give feedback is considered a resource saving by the university.  Future examiner training recommended.  Author recommends the use of clinical clerk examiners during low stakes, in-program evaluations for more junior colleagues. |
| Burgess et al, 2013  Senior medical students as peer examiners in an OSCE | **PA** | University of Sydney, Australia  Practice OSCEs | 40/98 (41%) of Year 4 medical students participated as examiners.  Voluntary participation.  All fourth year students invited to participate as assessors. | 105/115 (91%) of Year 2 students were examined (across a 2 year study). | A one-hour training session addressing their assigned OSCE station, marking criteria, examination and feedback techniques. | Five station practice clinical skills (hx & examination) & procedural skills OSCE. | Student examiners were highly confident in their ability to assess performance but were too lenient in providing a ‘global’ performance score. Further training in how to globally assess students’ overall performance objectively and provide accurate feedback is recommended.  Student examiners found peer assessment to be a very useful learning activity that  provided:   - Preparation for future careers - Development of professional attributes - Revision of knowledge and clinical skills |
| Burgess et al, 2012  Teaching skills for students: our future educators | **TT** | University of Sydney, Australia  ‘Teaching on the Run’ (TOR) program | 17/53 third year medical students participated.  Voluntary participation.  All third year students invited to participate via email. | NA | A dedicated teacher-training program consisting of six module training session x 3 hrs each; total 18 hrs (Titles of the modules not reported). | NA | Program highly valued by participants.  Participants perceived:   - Increased appreciation of educational theory and practice. - Increased ability to plan learning activities and provide effective feedback to their peers. - Increased value by senior academic staff. - Weakness in the assessment of their peers. |
| Brazeau et al, 2002 | **PF** | New Jersey Medical School, USA  Using OSCE as a teaching tool including peer feedback. | Year 3 students.  Compulsory participation.  Part of the teaching curriculum  Family Medicine Clerkship. | Third year students | No peer feedback training detailed. | Each student completed a 10 min OSCE station with standardized patients, observed via video by a group of six to eight students. Faculty facilitator then led a 12 min feedback to the student and nominated one student observer to give feedback. | The process of students giving feedback to their peers is seen as educationally usefully by faculty and by students.  No measurement on the accuracy of the student feedback was discussed. |
| Field et al, 2007 | **PT/**  **PF** | University of Glasgow Medical School, UK  Using PAL techniques to increase trainees’ exposure to clinical examination skills using the cardiovascular, respiratory and gastrointestinal systems. | Year 4 & Year 5 students.  Voluntary participation.  Students approached staff to do an elective option in teaching and learning techniques. | Eighty-six Junior year 1 & year 2 students.  Tutees were volunteers recruited by posters & email. | A dedicated training program included a literature review, watching training videos describing examination techniques and practicing these skills on each other under supervision. | 32 groups of up to 5 trainees (median 3) watched a clinical examination video; trainers explained the relevant techniques; trainees were video- taped practising examination; after feedback the procedure was repeated with guidance from trainers. | Trainees perceived that:   - Their confidence in examining the relevant system was high after the session - Trainers were well informed - They would all recommend the training to friends. - Trainers became more efficient across program iterations.   Trainers reported:   - More confidence in examination skills. - Better understanding of teaching techniques. - That they all valued the experience. |
| Glynn et al, 2006 | **PT** | National University of Ireland, Galway, Ireland  The Department of General Practice 6wk Early Patient Contact (EPC) program with a 2hr dedicated session on communication skills. | Two Year 5 students.  Voluntary participation.    Recruited via a short presentation about the PAL initiative made to the class. Tutors were then chosen if completed:  Communication skills and general practice modular training; summative assessment for that module; all communication OSCE stations and MCQ. | Fourteen Year 2 students in the EPC program who volunteered after an in-class presentation. | Not addressed | Peer Tutors facilitated a small group experiential communication skills session using role-play. | PAL was a resource saving strategy.  There was a high level of acceptance by both tutors and tutees.  Tutors and tutees described feeling a more relaxed & comfortable (i.e. ‘safe’) learning environment.  Tutees felt the tutors’ immediacy with the material and their student-like approach was advantageous.  The exchange of knowledge was viewed as the greatest limitation of PAL as tutors may not have been as knowledgeable as a faculty member. |
| Graham et al, 2008 | **PT** | University of Glasgow, Scotland.  Undergraduate rheumatology: PAL training in GALS (gait, arms, legs, spine) for musculoskeletal system (MSS) examination. | 12 senior students (from years 3-5) were trained in PAL and the GALS screening technique.  Voluntary participation.    19 students were recruited and trained by a physiotherapist. | Forty-five year 2 students volunteered for additional training in the use of GALS by PAL. Tutors recruited volunteers via posters in teaching sessions and year group email. | PAL training module in GALs consisted of: A literature review and training in educational theory and generation of a paper on PAL and small group teaching; Observation of the GALS video, practising techniques & critically reviewing performance under supervision; Submission of dissertation reflecting on PAL and the training program. | Two 2hr PAL training sessions. | Tutees that received additional training in GLS via PAL demonstrated no significant differences in confidence levels when compared with students who did not opt for additional training.  PAL was comparable with that delivered by a specialist. |
| Heckman et al, 2008 | **PT** | University-Hospital Erlangen, Germany  Peer-assisted clinical skills training for students during their neurology clerkship  Neurology clerkship lasts 1 week and takes place in the fifth year. | Year 5 students  Voluntary participation.  Tutors needed to have successfully passed the clerkship one semester earlier. | Year 5 medical students randomised to received clinical skills training by either peer (experimental group, n= 66) or experienced staff (control group, n=56). | Tutors trained in demonstrating the neurological examination and in instructing the lumbar puncture by an experienced neurologist. | Not reported | PAL was a financial resource saving strategy.  OSCE and written exams showed no significant difference in results between the experimental and control groups of students.  Self-assessed learning gain was equally rated in both groups. |
| Hudson and Tonkin, 2008. | **PT** | University of Wollongong, Australia  PAL in clinical skills training, comparing peer tutoring versus practitioner tutoring | Year 6 (n=29), final year students.  Voluntary participation.  Recruited via a flyer posted on student and online noticeboards or by personal contact at large-group sessions. Peer tutors taught 1 hour per week for 14 weeks. | Year 2 students (n=131) were randomly allocated to be taught peers or by paid doctors. | All tutors attended a training workshop and had ongoing content and educational support from the clinical skills director.  Some tutors tutored in pairs, and some individually. | A series of 14 small group structured tutorials where students received feedback on their history-taking and examination skills, using a simulated patient (SP). Small groups of 10-12 students. | Early clinical exposure and limited resources were reasons for investigating peer teaching.  Tutors reported personal benefits from teaching, including:   - A deeper level of understanding because they had to review to teach. - Fostered ability to admit uncertainty - Increased confidence   Both student cohorts achieved equally in clinical skills exams.  The PAL environment was perceived as comfortable by tutors and tutees and fostered confidence in participants.  Peer tutors created a more active learning environment than doctors. |
| Knobe et al, 2010. | **PT** | Aachen University, Germany  Ultrasound interpretation  using student teachers to teach musculoskeletal ultrasound (MSUS).  The program was part of a module on sports medicine | Nine Year 3 & 4    Voluntary participation.  Selected at random and invited via email. | Tutors & Tutees from same year group. Students allocated to either PAL group teaching delivered peers, or Staff group, where students were taught by doctors (with 6yr+ specialist experience). | 30 minute general training and 1 week of self-teaching.  30 minute introduction to the use of the ultrasound machine and one week of preparation time with the appropriate background literature. In addition, student tutors were able to access information from the European League against Rheumatology using the internet. | Small group teaching (6-14 students) Students were taught in two lessons, each lasting 120 minutes. The first 30 minutes covered theoretical background. Students then participated in a 90-minute practical session in which they were able to practise imaging | Resource saving as students had greater access to teachers without the added cost of professional staff.  There was no difference in performance between peer and staff tutor groups  The student tutors:   - Showed significantly better results over all in their MCQ and OSCE examination. - Were confident in their capability and rated their learning success highly. - Were willing to tutor again in the future. - Felt that the skill set required to teach the shoulder ultrasound did not require the use of an experienced doctor. |
| Merglen et al, 2008 | **TT** | Institution unknown  Peer teaching in clinical learning: A student-initiated, undergraduate, peer teaching skills program  Faculty and students gathered a multi-professional teaching team of varying experience (from student to professor) and profiles to teach the program. | 16 elective Year 4 students in a 6-year curriculum.  Voluntary participation. | NA | A dedicated elective teaching program. 5x 2hr interactive workshops addressing six themes: one- to-one peer teaching; training in procedural skills; giving feedback; orienting peers in a new clinical environment; interprofessional communication, and reflective practice based on logbook recordings. | NA | Students expressed high levels of program satisfaction.  Its potential impact on preparing future residents’ teaching skills requires further research |
| Nestel & Kidd, 2005. | **PT** | Imperial College London, UK  PAL in patient-centred interviewing  A communication program spans the entire curriculum. In the first year, students complete five sessions focused specifically on communication. | Twenty-one Year 3 students.  Voluntary participation.  All third-year students invited to participate via an in-lecture description and electronic posting. | One third of the first year cohort. | Student tutors attended a three-hour preparatory workshop to develop facilitation skills and review content, structure and process of the interviewing a simulated patient session. Students set their own learning objectives in addition to those set out by the facilitators. Teaching-learning strategies used included: brainstorming, discussing, reflective writing, role-play, reviewing videotapes and a workshop manual (Nestel & Kidd, 2002). | In the fifth program session (PAL implementation session), students interview a simulated patient and then receive feedback on their patient-centered interviewing skills from the patient, other first-year students and the facilitator.  2:6 tutor to student ratio in a small group workshop. | Subjective data demonstrated some benefits for student tutors as a direct consequence of participating in the PAL project. However, the objective measures did not identify benefits.  The self-report data on facilitation suggests that students gained knowledge and skills during the project.  Student tutors seemed to benefit in terms of raising their awareness of communication but knowledge does not necessarily lead to improved skill demonstrated in the absence of change in the interviewing skills of student tutors. |
| Nikendei et al, 2008 | **PT** | University of Heidelberg Medical Hospital, Germany  Internal medicine PAL program to support the transfer of knowledge and skills from lectures, PBL lessons, clinical skills & communication training sessions directly to the bedside. | Final Year students.  Recruitment of up to 24 final year students per year as peer tutors during their introduction week. (Does not specify the recruitment process) | Third year students (n=320). During a 5wk internal medicine ward placement, students take part in 10 PAL sessions. | Tutors trained for the assignment as peer tutors working from a tutor manual and were supervised on a weekly basis by an experienced medical tutor. All received a financial allowance. | Small groups of 3 - 8. Tutorials primarily at the bedside, & additional weekly tutor/student meetings possible and expressly desired. | Peer tutors acknowledge that participating in the program results in an enhancement of personal teaching skills and an increase in basic clinical skills and also affirm their willingness to serve as a peer tutor in the future. |
| Raupach et al, 2010 | **PT** | University Hospital Gottingen, Gottingen, Germany  A 6-week cardiorespiratory course: Three introductory ECG lectures in which basic principles were introduced, followed by eight sessions of either traditional or small-group, near-peer teaching | Near peer tutors were students one year ahead of learners.  Recruitment method not discussed. | Year 4 medical students (n = 335) randomised to receive either eight traditional ECG lectures or the same amount of small-group, near-peer teaching.  Participation in all sessions was voluntary, but attendance was recorded for each student. | Thorough training covered: self-study, presenting short talks on important aspects of ECG interpretation, and the principles of small- group teaching. Peer teachers produced interpretations of all ECGs contained in the interpretation guide and these were discussed and agreed upon in the presence of an ECG expert. | Small-group sessions of 8-9 students. Students studied 4-5 ECG tracings during which peer teachers were advised to facilitate discussion rather than providing explanations and correct interpretations. | No reference to peer teacher specific outcomes. Overall, the study concluded “Small-group peer teaching appeared at least as effective as traditional lecture-based expert teaching in generating learning effort and learning effects in ECG skills”. |
| Robinson et al, 2010 | **PT/TT** | University of Bristol, UK.  PAL in clinical skills of performing an episiotomy and its repair.  Reproductive Health and Care of the Newborn (RHCN) obstetric program | Two 4th year medical students were randomly recruited from the undergraduate program and training incorporated into the RHCN program. Of these two student-tutors one provided the peer-assisted training and the other acted as a peer-assessor. | Tutees were recruited from the 4th year Bristol University medical student RHCN program and randomly allocated to a peer teaching (n=5) or trainee obstetrician group (n=3). | Both peer-tutor and peer-assessor were taught PAL techniques, the principles of adult learning, and learner-centered teaching models, and trained in the principles and clinical skills of performing an episiotomy and its repair. The techniques were practiced with one-to-one supervision by two trainee obstetricians. | Tutees taught within a structured program, including: a lecture-based (power-point) presentation; a video on the principles of episiotomy, knot tying and suturing techniques; & practice of episiotomy and its repair using a model. | Trainee perception of tutor knowledge did not significantly vary between the groups and knowledge results were positive for both groups.  The peer tutor enjoyed acquiring teaching skills. |
| Silbert & Lake, 2012  Peer-assisted learning in teaching clinical examination to junior medical students | **PT/TT** | The University of Western Australia, Australia.  Student Grand Rounds (SGR) for teaching clinical examination to junior medical students. Developed and run entirely by students without faculty involvement. SGR provides extracurricular clinical examination tutorials utilising peer physical examination (PPE) and hospital patients. | Senior Medical students (year 4-6).  Voluntary participation.  A senior medical student elected on an annual basis into the role of SGR coordinator by the medical student body. | Senior medical students (in years 4–6) teach their junior peers.  Tutorials were specifically linked to the formal teaching in year 3 of the medical course. | Student tutors attend a modified Teaching on the Run (TOTR) course consisting of two 3hr small group interactive workshops (‘planning to teach’ and ‘teaching’). Outcomes included planning a session, defining outcomes, optimising learning, involving patients, giving feedback, self-evaluation and managing group dynamics. | Sixty min sessions, with average 5:1 students to tutor ratio.  PAL session:  1) Examination technique sessions: students practiced examinations on each other under the supervision of the tutors, or  2) ‘Signs round’ sessions: students practiced examinations on real patients in teaching hosp, to familiarise them with patient interaction & to elicit clinical signs. | Participant impressions of the workshops were highly positive and all intended workshop outcomes were achieved.  Tutors considered all PAL tutorials to be beneficial, both to themselves and to their students.  Tutors reported improvement in their own knowledge, teaching and feedback skills. They also reported incorporating techniques from TOR sessions. |
| Weyrich et al, 2008 | **TT/PT** | University Hospital of Tübingen, Germany  Undergraduate technical skills training guided by student tutors. Basic procedural skills in internal medicine | 14 Year 4 and 5 students  Voluntary participation.  Personal interview with mentoring doctor.  Former experiences as a tutor, teacher, team leader, accomplished clerkships as well as the level of motivation served as criteria for selection, with tutors receiving a contract and financial compensation from the faculty as student assistants. | Participation was obligatory for all 3rd year medical students. | A consultant of internal medicine and six 4th year students met in a focus group to assess the training syllabus.  Two x 3hr consultant-led training sessions carried out in the skills lab of internal medicine. Former clerkships were discussed to reach a consensus concerning how each skill should be taught. All procedures were demonstrated in a step-by-step manner by consultants and repeatedly practiced by the tutors. Additional clinical background information was provided for each of the technical skills. A manual compiled by five experienced clinicians was supplied to the peer tutors as a reference guide. | Training involved groups of 6-8 tutees facilitated by 2 tutors (4:1). Two x 3hr skills lab sessions (one week apart). Tutors taught the background information according to the previous training sessions with the consultants and were instructed to adhere to the tutor manual provided. Tutees were given the opportunity to practise the skills under supervision of the tutors with continuous feedback. In case of uncertainties or questions, it was possible to localize the contact doctor by pager at any time. | The tutors:   - Noted a pleasant learning atmosphere. - Appreciated the constant availability of a contact consultant in case of problems or questions. - Acknowledged that their tutoring role had helped them to overcome previous anxiety and self-doubt (n=3). - Agreed that the pleasure of being accepted as a teacher in addition to the observed gain in their group leadership qualities enhanced personal motivation. They considered this profit to far exceed any financial compensation and would act as a tutor again even without payment. |
| Bucknall et al, 2007 | P  PA | University of Birmingham, United Kingdom  Students undergoing an end-of course test in basic life support were simultaneously assessed by peer & faculty assessors.  162 students were assessed by 9 sets of peers and faculty examiners.  Methods:   - Questionnaire to measure student’s & peer assessor’s attitudes to peer assessment. - Reliability of assessment results. | Voluntary  9 fourth year medical student volunteers | 9 4^th^ yr medical students assessed first year medical, nursing & physiotherapy students, during | 9 fourth year medical student volunteers werer trained to become peer assessors by attending a European Rescuscitation Council Basic Life Support and Automated Externa Defibrillation instructor course. This on e day course trined participants in teaching and assessment skills. After training, the students worked with experienced faculty BLS assessors for 4 hours to consolidate their assessment skills. |  | - Accuracy in marking   Student perception of peer assessment.  Results:  Student examiner marking was regarded as reliable, with a good level of agreement with faculty examiners.  Peer assessors were more cautious of awarding a pass than faculty.  Students preferred peer assessment. |
| 21. Perera et al, 2010 | **PF** | International Medical University, Kuala Lumpur, Malaysia  Objective structured self-assessment and peer feedback (OSSP) incorporated into small group communication skills teaching | Same year group. | Semester one medical undergraduate program students (n=202) divided using academic entrance grades into experimental (peer feedback & self assessment) & control (teacher instructed) groups. | The experimental group was trained on provision of feedback to peers and self-assessment at a mock role-play session conducted by two teachers who were trained and allocated for instructing the experimental group. The students had an opportunity to practice the same after the teacher role-play sessions. | SPs role played and provided feedback to the student doctor; the student doctor completed a structured self-assessment tool containing open ended and closed questions for reflection; peers then provided feedback to student on any additional points with the aid of the same log sheet. | OSSP was significantly effective in meeting performance goals for interview skills. Results suggest that students who are exposed to self assessment and peer feedback in a small group practice based setting may learn better than students who receive only SP and tutor feedback. A small percentage felt either shy, uncomfortable, or harassed frequently, when others were providing feedback. Only 9.6% felt awkward frequently while providing feedback to colleagues. |

PT= Peer Teaching, PF = Peer Feedback, TT = Teacher Training, PA = Peer Assessment
